# Auditory Mismatch Negativity and Repetition Suppression Deficits in Schizophrenia Explained by Irregular Computation of Prediction Error

**DOI:** 10.1371/journal.pone.0126775

**Published:** 2015-05-08

**Authors:** Johannes Rentzsch, Christina Shen, Maria C. Jockers-Scherübl, Jürgen Gallinat, Andres H. Neuhaus

**Affiliations:** 1 Department of Psychiatry, Charité University Medicine, Berlin, Germany; 2 Department of Psychiatry, Oberhavel Hospital, Hennigsdorf, Germany; 3 Department of Psychiatry, University Hospital Eppendorf, Hamburg, Germany; University of Jyväskylä, FINLAND

## Abstract

**Background:**

The predictive coding model is rapidly gaining attention in schizophrenia research. It posits the neuronal computation of residual variance (‘prediction error’) between sensory information and top-down expectation through multiple hierarchical levels. Event-related potentials (ERP) reflect cortical processing stages that are increasingly interpreted in the light of the predictive coding hypothesis. Both mismatch negativity (MMN) and repetition suppression (RS) measures are considered a prediction error correlates based on error detection and error minimization, respectively.

**Methods:**

Twenty-five schizophrenia patients and 25 healthy controls completed auditory tasks designed to elicit MMN and RS responses that were investigated using repeated measures models and strong spatio-temporal a priori hypothesis based on previous research. Separate correlations were performed for controls and schizophrenia patients, using age and clinical variables as covariates.

**Results:**

MMN and RS deficits were largely replicated in our sample of schizophrenia patients. Moreover, MMN and RS measures were strongly correlated in healthy controls, while no correlation was found in schizophrenia patients. Single-trial analyses indicated significantly lower signal-to-noise ratio during prediction error computation in schizophrenia.

**Conclusions:**

This study provides evidence that auditory ERP components relevant for schizophrenia research can be reconciled in the light of the predictive coding framework. The lack of any correlation between the investigated measures in schizophrenia patients suggests a disruption of predictive coding mechanisms in general. More specifically, these results suggest that schizophrenia is associated with an irregular computation of residual variance between sensory input and top-down models, i.e. prediction error.

## Introduction

Recently, the predictive coding hypothesis has been proposed as a unifying theory of cortical computational function [1]. This theory posits the neuronal computation of residual variance (‘prediction error’) between sensory information and top-down expectation through multiple hierarchical levels. In order to obtain a realistic model of the environment, the human brain then seeks to minimize the prediction error.

Predictive coding has become a dominant theory in the reward and salience processing literature [2–4]. Regarding schizophrenia, recent research has associated predictive coding deficits with deficient reward [5] and salience processing [6]. Moreover, the predictive coding theory has been successfully applied to psychopathological phenomena, such as delusional thoughts [7], auditory hallucinations [8], passivity experience [9], and altered sense of agency [10]. Finally, sensory learning processes are also increasingly interpreted in the light of predictive coding [11,12].

Event-related potentials (ERP) reflect cortical processing stages that are increasingly interpreted in the light of the predictive coding theory, which is especially true for the mismatch negativity (MMN) and repetition suppression (RS) measures [1]. The MMN component signals the perceived difference between a frequent standard stimulus (creating an expectation) and a rare deviant stimulus (violating this expectation). This mechanism is compatible with cumulating evidence that MMN may be a direct correlate of the prediction error [13,14]. On the other hand, RS measures are based on adaptation processes following repeated stimulus presentation, in the auditory modality also referred to as ‘gating’. Stimulus repetitions increase our confidence by confirming internal top-down models and thereby reducing prediction error; consequently, neuronal signal differences are thought to reflect similarity between stimuli which is compatible with the concept of minimized prediction error signals [1].

Given that MMN reflects the presence and RS reflects the reduction of prediction error signals, we reasoned that both measures should be strongly correlated in controls. Taking into account increasing evidence for prediction error deficits in schizophrenia [5,6,15], we hypothesized that the same correlation should be considerably lower in schizophrenia. Auditory MMN and RS measures were thus investigated with a strong a priori mechanistic hypothesis. Given long-standing evidence from a large number of studies for deficits of both MMN [16–18] and auditory ‘gating’ [19–21] and other repetition suppression (e.g. ‘roving’) measures in schizophrenia [22,23], this approach is suitable to increase our understanding of these frequently reported deficits on a mechanistic level.

A few studies have investigated the relationship of the repetition suppression of auditory evoked responses and the mismatch negativity, however, these studies yielded inconsistent findings with partially positive [24,25], and negative results [26]. The present study therefore aimed at re-evaluating the potential correlation between repetition suppression and MMN with sufficient statistical power.

## Methods and Materials

### Participants

Twenty-five schizophrenia patients (10 female) were included in this study. They met DSM-IV criteria for schizophrenia and had no psychiatric disorder other than schizophrenia and nicotine abuse/dependence. A urine drug screening was carried out on the day of recording; a positive test result led to exclusion from the study. None of the included patients had a history of severe medical disorder or severe neurological disorder. All patients were recruited from the Department of Psychiatry, Charité University Medicine, Berlin.

Twenty-five healthy subjects (10 female) recruited via newspapers were included in this study. Controls were matched for age, sex, education years, and IQ (in case of schizophrenia estimated pre-morbid IQ). Controls were screened for mental and physical health and were excluded when meeting the criteria of psychiatric disorders according to DSM-IV as determined by structured clinical interviews (except for nicotine abuse/ dependence). Further reasons for exclusion were a family history of psychiatric illness, medical or neurological disorders, or intake of psychotropic drugs.

Smoking as usual was allowed up to one hour before testing to avoid any withdraw symptoms (time between arrival and starting of recording lasted at least one hour). Subjects underwent a urinary drug screening to exclude current use of drugs (Mahsan-Kombi-DOA4, Reinbek, Germany). Table 1 summarizes demographic and clinical variables for all participants. All participants were right-handed and had normal audition.

**Table 1:** Demographic and clinical variables as mean ± SD (range).

	Schizophrenia (N = 25)	Control (N = 25)
Age [years]	29.72 ± 7.9 (18–47)	29.52 ± 7.9 (18–48)
Education [years]	11.68 ± 1.7 (8–15)	12.50 ± 1.5 (8–15)
Premorbid IQ^1^	112.00 ± 18.82	116.08 ± 16.4
N smokers^2^	15	6
Cigarettes/day^3^	21.2 ± 4.6	8.8 ± 5.1
Duration of illness [years]	5.20 ± 4.6	--
Number of hospitalizations	2.6 ± 2.0	--
CPZ equivalents [mg/d]	439.84 ± 260.2	--
PANSS total score	64.11 ± 16.6	--
PANSS positive subscale	12.61 ± 3.1	--
PANSS negative subscale	19.17 ± 6.1	--
PANSS general subscale	32.33 ± 9.4	--

Abbreviations: CPZ, chlorpromazine; IQ, intelligence quotient; PANSS, Positive And Negative Syndrome Scale. There were no significant between-group differences regarding age, education, or estimated pre-morbid IQ.

^1^Pre-morbid IQ was estimated using a multiple choice vocabulary test.

^2^Chi^2^ = 6.7, p< .01.

^3^ Average cigarettes smoked per day in the last six months in the group of smokers; df = 17, t = 5.3, p<.00006.

### Ethics

The capacity to consent to the study was assessed by the attending psychiatrist and by author JR according to the principles defined by Kröber [27]. Patients who were unable to consent or were hospitalized by law were not included in the study. Following screening, patients were given the possibility to consult persons of trust before participating in the study. Following this procedure, written informed consent was obtained by all controls and patients before participating. The study was approved by the institutional ethics committee of the Charité University Medicine and conducted in accordance with the Declaration of Helsinki.

### Procedure and task design

Subjects were seated in a chair with a head rest in a sound-attenuated and electrically shielded room. Auditory stimuli were presented binaurally by calibrated headphones. Following recommendations for MMN recording [28], we employed two passive auditory paradigms while the participants watched a silent cartoon. In the MMN protocol, 900 stimuli were presented in total, consisting of standards (frequency 1,000 Hz; duration 80 ms; probability .9) and deviants (frequency 930 Hz; duration 80 ms; probability .1) that were applied with an inter-trial interval between 350 and 650 ms. RS stimuli consisted of 100 pairs of identical click stimuli (1-ms square waves; 500 ms stimulus onset asynchrony) with an inter-trial interval of 3,400 ± 727 ms. All stimuli were presented at 80 dB sound pressure level. Between the two paradigms, there was a short break of 2 min.

### EEG data acquisition and analysis

EEG was collected with 32 Ag/Ag-Cl electrodes according to the extended international 10/20 system using an electrode cap. Additional electrodes were placed at left and right mastoids and at the outer canthus of the left eye. A ground electrode was placed on the forehead. Electrode impedances were kept below 10 kΩ. All channels were internally referenced to Cz. EEG was recorded with a Neuroscan SynAmps (El Paso, TX, US) and continuously digitized at a sampling rate of 500 Hz. During acquisition, EEG data were high-pass filtered at 0.016 Hz.

Offline ERP analysis was conducted with Brain Vision Analyzer 2.0 (Brain Products, Munich, Germany). Ocular and cardio-electric artifact correction was performed using an independent component analysis approach [29]. Data were then re-referenced to common average and digitally filtered (MMN, N100, P200: 0.5–30 Hz; P50: 10–70 Hz, additional notch filter at 50 Hz; butterworth filters, 24 dB). Remaining artifacts (80 μV at any electrode) were marked for later removal. Data were segmented according to stimulus class and relative to stimulus onset (MMN: -150 to 400 ms; P50/ N100/ P200: -150 ms to 800 ms after first click stimulus). After final automated artifact rejection and baseline correction, the remaining trials were averaged per subject and experimental condition (standard; deviant; first click; second click).

Individual ERP averages were used to construct the MMN component by subtracting the waveforms elicited by standards from those elicited by deviants. MMN amplitudes were defined as the most negative peak between 90 and 210 ms according to the grand average. The amplitudes of the event-related potentials elicited by click-pair stimuli were defined as the highest amplitude in the time range 50–75 ms (P50), 70–130 ms (N100), 130–250 ms (P200) after either the first or second stimulus. RS was computed as the amplitude difference in response to the first and second stimulus; this approach is also in line with the better reliability of difference compared to ratio measures [30]. For all components, Fz and Cz were analyzed, corresponding to their individual scalp topography.

### Statistical analysis

Statistical analyses were done with IBM SPSS version 19 (Armonk, NY, US). Demographic data were analyzed with t-tests for independent samples or chi-squared tests, as appropriate. Electrophysiological parameters, i.e. MMN and RS measures of P50, N100, and P200 components were analyzed with repeated measures analyses of variance (ANOVAs). For each ANOVA, we entered ‘electrode’ (Fz vs. Cz) as within-subjects factor and ‘group’ (schizophrenia vs. control) as between-subjects factor. For all ANOVAs, partial eta squared (η^2^) served as an estimate of effect size, i.e. the proportion of variance accounted for by the model. Correlation analyses were done using Pearson rank correlations using age and clinical variables as covariates. All tests were done as two-tailed tests with an alpha level of p< .05.

## Results

### MMN paradigm

**Fig 1:**
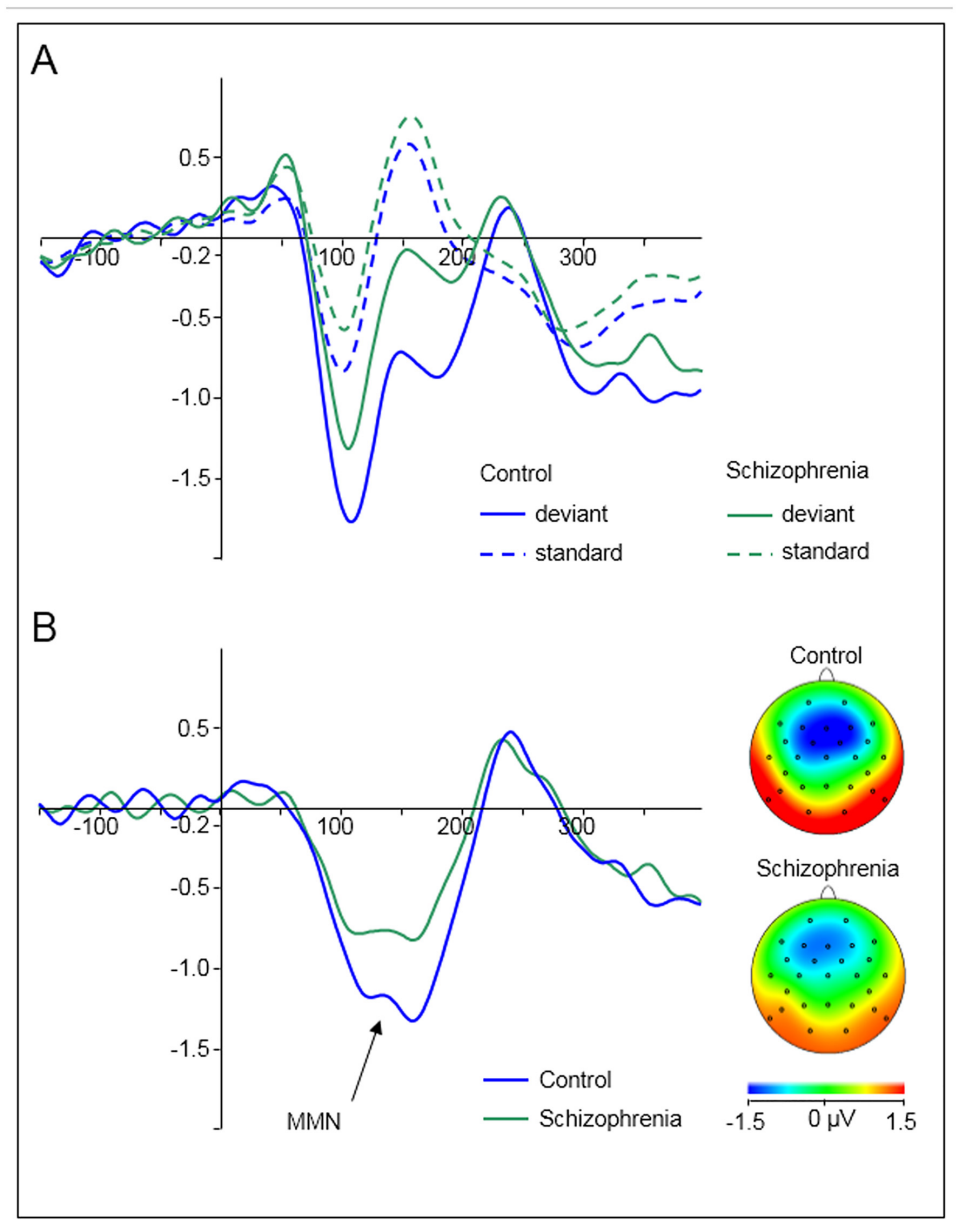
Grand averaged auditory evoked responses of healthy controls (blue) and schizophrenia patients (green) pooled across electrodes Fz and Cz. Stimulus onset at 0 ms. (A) Event-related responses to deviant (solid lines) and to standard stimuli (dotted lines) stratified by group. (B) Difference waveforms (deviant minus standard) with a clear mismatch negativity component between 100 and 200 ms. Topographic maps are given for the maximum amplitude of the MMN for both groups.

ANOVA revealed significant main effects of the factors ‘electrode’ and ‘group’. The effect of ‘electrode’ was based on significantly larger, i.e. more negative, MMN waveforms at Fz (-1.81 ± 0.9 μV) compared with Cz (-1.09 ± 1.0 μV; F(1,48) = 32.071; p< .001; η^2^ = .401). The effect of group was based on larger MMN waveforms in controls (-1.70 ± 0.9 μV) compared with schizophrenia patients (-1.19 ± 0.6 μV; F(1,48) = 5.023; p = .03; η^2^ = .095; see Fig 1). Crucially, there was no interaction of ‘electrode’ x ‘group’, indicating that between-group differences were not significantly different between electrodes.

### Repetition suppression paradigm

**Fig 2:**
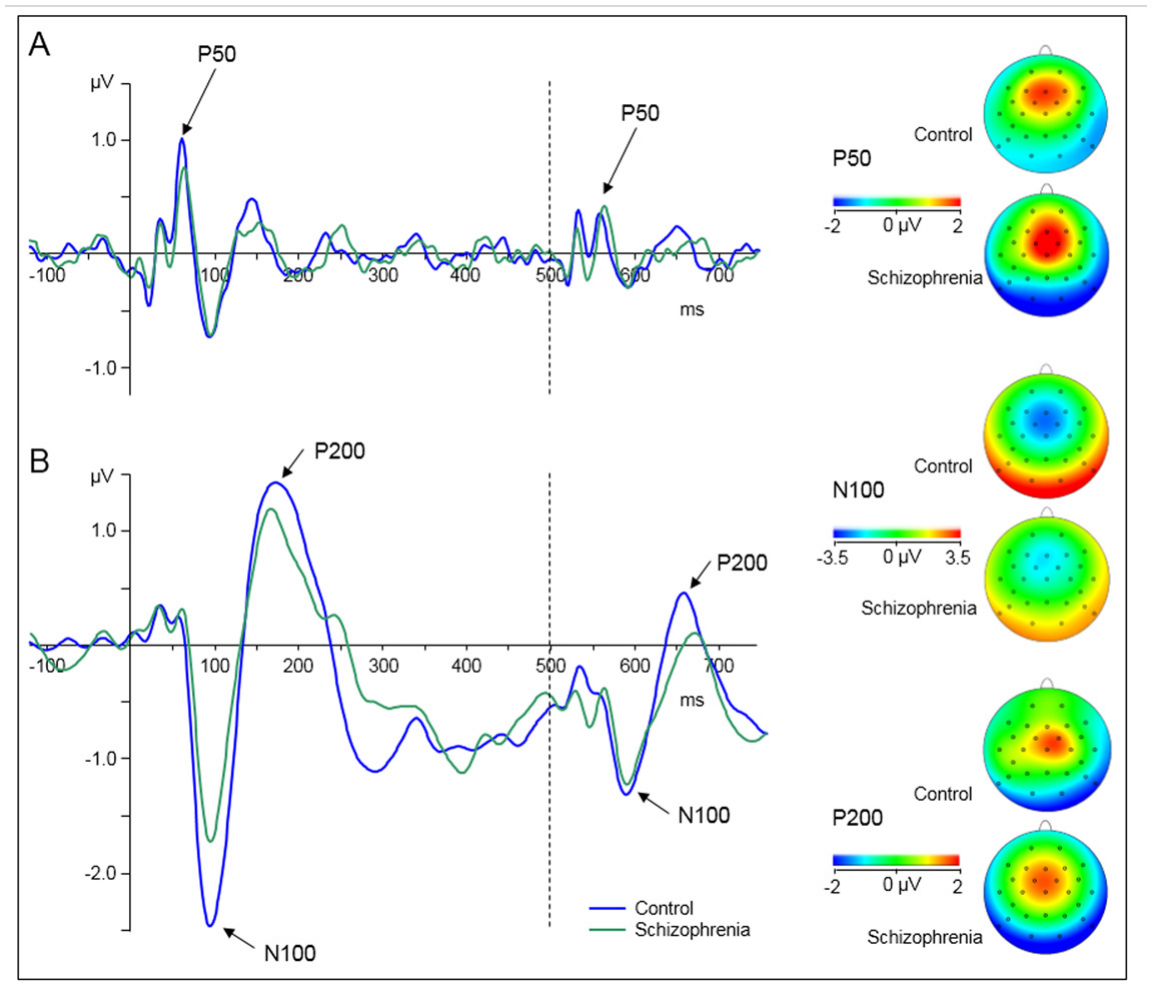
Grand averaged event-related potential waveforms elicited by auditory click-conditioning stimuli for healthy controls (blue line) and schizophrenia patients (green line) pooled across electrodes Fz and Cz. Onset of stimuli is at 0 ms (first click, solid line) and at 500 ms (second click, dashed line). Click-conditioning stimuli evoke a P50 component (A) and a N100/P200 component complex (B). Topographic maps are given for the maximum amplitude of the P50, N100 and P200 amplitude elicited by the first click stimuli for both groups.

We analyzed three consecutive ERP components associated with auditory RS, i.e. P50, N100, and P200. For the P50 measure, ANOVA indicated no significant main effects or interactions. The N100 measure was associated with a main effect of ‘group’, where controls showed significantly greater amplitude suppression in response to the repeated stimulus than schizophrenia patients (-1.75 ± 1.3 μV vs. -1.00 ± 1.1 μV; F(1,48) = 4.973; p = .03; η^2^ = .094; see Fig 2a). Similarly, P200 showed a significant main effect of ‘group’ that was based on greater suppression of the P200 response to the repeated stimulus of controls compared with schizophrenia patients (2.50 ± 1.9 μV vs. 1.54 ± 1.3 μV; F(1,48) = 4.442; p = .04; η^2^ = .085; see Fig 2b).

### Correlation analysis

**Fig 3:**
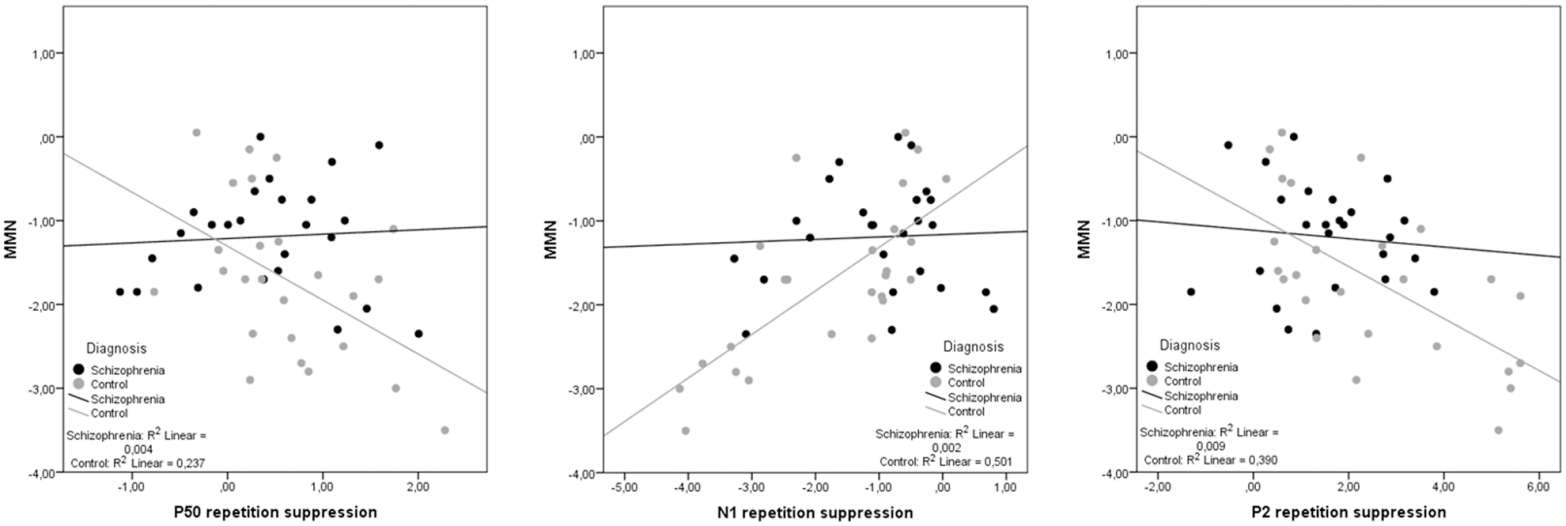
Scatterplots of mismatch negativity (MMN) and repetition suppression measures of P50, N100, and P200. Both MMN and repetition suppression measures are conceptualized as event-related components that signal the difference between predicted and unexpected stimuli (i.e. prediction error). String correlations are therefore expected, which are present in healthy controls (grey dots), but absent in schizophrenia patients (black dots).

ERP components were averaged across electrodes due to the failure to statistically determine different between-group differences at different electrodes and in order to reduce the number of analyses that might prove circular in nature [31]. Hence, pooled MMN amplitudes were correlated with RS measures separately for each group. In controls, MMN was correlated with RS indices of P50 (r = -.49; p = .014), N100 (r = -.71; p< .001), and P200 (r = -.62; p = .001). Very similar correlations were obtained when using age as a control variable in partial correlations of MMN with RS indices of P50 (r = -.45; p = .029), N100 (r = -.70; p< .001), and P200 (r = -.61; p = .002). No significant correlation was obtained in schizophrenia patients (MMN/ P50, r = .07, p> .7; MMN/ N100, r = -.05, p> .8; MMN/ P200, r = -.1, p> .6), even when controlling for PANSS total score (MMN/ P50, r = .2, p> .3; MMN/ N100, r = -.07, p> .7; MMN/ P200, r = -.08, p> .7) or number of cigarettes per day (MMN/ P50, r< -.01, p> .9; MMN/ N100, r = .04, p> .8; MMN/ P200, r = -.1, p> .6) in partial correlations. In contrast, partial correlations with daily cigarette consumption as covariate remained significant in healthy controls (P50: r = -.45, p< .03; N100: r = .71, p< .0001; P200: r = -.65, p< .001). The correlation group differences between MMN and repetition suppression parameters were significant (P50: z = -4.4, p< .01; N100: z = -2.7, p< .01; P200: z = 2.1, p< .05). Fig 3 illustrates Pearson correlations.

## Discussion

According to the increasingly influential predictive coding hypothesis as a potentially universal theory of cortical computational mechanisms, neuronal signaling serves the purpose of reducing surprise, i.e. prediction error. The present study investigated whether auditory ERP components comply with this basic assumption of predictive coding. Specifically, we investigated auditory MMN and RS measures as surrogate markers of the prediction error. According to our hypotheses, strong correlations between these ERP measures were found in controls while there was no such correlation in schizophrenia patients; partial correlations did not indicate a significant confound by age, PANSS, or smoking. These results are consistent with previous hypotheses on cortical signalling within the predictive coding framework.

On a mechanistic level, the value of this study is constituted by normative data that reconcile two ERP measures—previously thought of to be unrelated—into a coherent predictive coding framework and by data of schizophrenia patients that indicate disrupted prediction error computation. Three studies have investigated the relation between repetition suppression and MMN so far. Gjini et al. [24] investigated MMN responses to frequency, duration, white noise, and ‘abstract’ (rare descending deviant tones among ascending standards) as well as P50, N100, and P200 repetition suppression in two relatively small samples of schizophrenia patients and controls (both N = 12). They found correlations between ‘abstract’ MMN and repetition suppression of P50 and P200 amplitudes in controls, but not schizophrenia patients; other measures, such as frequency MMN, duration MMN, white noise MMN, or N100 were not significantly correlated. Similar to our results, Kisley et al. [25] found a co-variation of the P50 repetition suppression and MMN amplitude in healthy individuals, but they did not investigate schizophrenia patients. Turetsky et al. [26] investigated several ERP measures including repetition suppression of P50 and N100 amplitudes as well as MMN. Before correlating those measures, however, ERP components were transformed using an oblique factor, which renders the negative correlations obtained in this study somewhat difficult to compare with our present results. In the light of these findings from previous studies, the present study clearly confirms an association between repetition suppression and MMN measures that dissociates with diagnosis.

Our results corroborate that schizophrenia is associated with a disruption of predictive coding function that is reflected in both reduced MMN and RS. Our data confirm previous findings of deficits of both MMN [16–18] and auditory ‘gating’ measures [19,32] in schizophrenia. However, an RS deficit in schizophrenia was only found for late but not for middle-latency evoked potentials. It remains unclear whether the atypical antipsychotic medication in our patient group caused a normalization of the P50 RS deficit as was reported earlier [33,34]. From a functional perspective, it has been previously suggested that ‘gating’ reflects the inhibition of irrelevant sensory input in terms of stimulus evaluation and salience detection, while MMN represents the pre-attentive response to violations of sensory memory traces [35–37]. Following these popular mechanistic views and given the similar magnitudes of the correlations found in this study, it might be speculated that echoic memory (MMN) is tightly related to salience detection at all stages of attention investigated here, i.e. according to the respective components' latencies (P50, N100, P200). A more persuasive explanation, however, would postulate a common computational mechanism that integrates our findings into a single coherent framework.

We therefore postulate correlations of other, yet not primarily related functional and neurocognitive domains that are claimed to be deficient in schizophrenia, due to a universal computational mechanism of cortical function, as suggested by the predictive coding theory. So far, however, no study has specifically assessed correlations between neuronal signals across these domains, i.e. reward and salience processing, auditory processing, or sense of agency. The present study strongly suggests that deficits across the aforementioned domains may be elegantly explained with a single computational deficit. This approach thus offers the opportunity to explain a variety of previously dispersed findings, which may considerably aid the field in focusing on specific research questions that could provoke fundamental answers across sensory and cognitive domains. The predictive coding account may provide a coherent model of cognitive dysfunction and its anatomical fundament in schizophrenia that is able to reconcile disconnection theory e.g. [38,39], computational models e.g [40,41], and functional imaging of cognitive deficits e.g. [42].

Limiting the results of study, we assessed a heterogeneous patient sample with differences in, e.g., age, symptom severity, chronicity, and medication. On the other hand, sample heterogeneity increases the ecological validity of the findings obtained. An interesting approach for future studies would be to assess cross-modal and cross-domain prediction errors in samples at risk versus first manifestation versus chronic patients to answer the question whether predictive coding deficits are present before onset, progress over time, or both. Previous studies consistently suggest that both MMN [43–45] and RS deficits predate the onset of illness [46,47]. An early deficit across these and other cognitive domains, however, has not been assessed. Next, our main result of differential MMN/ repetition suppression correlations between the two groups may have been facilitated by the data structure. The variance of the schizophrenia ERP data was considerably lower than in the control group, which might have obscured the detection of a correlation. This shortcoming cannot be corrected post hoc, but must be addressed in a separate study. Finally, although we assessed oddball and MMN measures, our approach does not allow for inferring causality. Our data are consistent with, but do not prove, the predictive coding nature of neuronal stimulus responses, as outlined by Friston [1]. Future studies could, at least partially, overcome this limitation with the additional assessment of an equal probability control condition e.g. [48] that would allow for differentiating, whether responses in the oddball condition reflect a predictive coding mechanisms or mere stimulus rarity.

In conclusion, the current study presents clear evidence for a functional mechanism that connects two sensory domains. The correlated neuronal signals can—at least from today’s perspective—only be accounted for by the predictive coding theory. The aberrant neuronal signals in schizophrenia are most likely the consequence of a deficient prediction error computation. Further pursuing this line of research may reveal answers of fundamental questions that are at the core of cognitive dysfunction in schizophrenia.
